# Experimental and Numerical Investigation of the Fracture Behavior of Extruded Wood–Plastic Composites under Bending

**DOI:** 10.3390/polym16111485

**Published:** 2024-05-23

**Authors:** Almontas Vilutis, Vytenis Jankauskas

**Affiliations:** Department of Mechanical, Energy and Biotechnology Engineering, Vytautas Magnus University, K. Donelaičio St. 58, LT-44248 Kaunas, Lithuania; almontas.vilutis@vdu.lt

**Keywords:** load-line displacement, crack propagation, fracture toughness, failure mode, numerical modeling, von Mises stress, stress triaxiality, Lode angle parameter

## Abstract

The ability of wood–plastic composites (WPCs) to withstand various loads and resist plastic failure is attracting more and more interest due to the global increase in demand for WPCs by over 6 million tons per year. Among the most important and innovative research methods are those based on fracture mechanics—their results enable material designers to optimize the structures of these hybrid polymer composites at the nano, micro and macro levels, and they allow engineers to more accurately evaluate and select functional, sustainable, long-lasting and safe product designs. In this study, standard single-edge notched bending (SENB) tests were used to analyze the fracture toughness of two different extruded WPCs along the longitudinal (L) and transverse (T) directions of extrusion. In addition to their resistance to crack propagation, critical fracture criteria, initial contact stiffness, fracture parameters and fracture surfaces, the mechanical properties of these composites were also investigated. The results showed that WPC-A coded composites withstood higher loads until failure in both directions compared to WPC-B. Despite the larger data spread, both types of composites were more resistant to crack propagation in the T direction. Mode II of crack propagation was clearly visible, while mode III was not as pronounced. The experimental results and the numerical finite element (FE) model developed up to 58% of the maximum load correlated well, and the obtained deformation curves were best approximated using cubic equations (*R*^2^ > 0.99). The shear stress zone and its location, as well as the distribution of the equivalent stresses, had a major influence on crack propagation in the fracture process zone (FZP).

## 1. Introduction

For many years, design decisions were influenced by the choice of materials available, and it makes sense that historical eras—the Stone, Bronze, and Iron Ages—were named after them. The situation has changed rapidly since the second half of the 20th century, as plastics and various composite materials started to be used in addition to the predominant metals. Nowadays, an engineer can choose from a variety of materials for a product design, ranging from 40 to 80 thousand [1]. Plastics are the third most commonly produced material after steel and concrete, and account for more than 11% of the municipal solid waste (MSW) stream [2]. One way to reduce this volume is to produce WPCs.

A projected annual volume of 7 million tons by 2025 [3] and a compound annual growth rate (CAGR) of 11.6% between 2023 and 2030 [4] indicate rapid growth in the demand for engineered wood–plastic composites. Interest in these materials, their properties and their potential applications has increased significantly. A significant market growth of over 10% is also forecast for all three of the thermoplastics that are commonly used in WPCs (PE, PP, PVC). The demand for sustainable building materials has also increased globally [4]. The latter are bio-based or biodegradable materials, the use of which contributes to solving the important problems of waste, environmental protection from pollution and reducing greenhouse gas emissions and the consumption of fossil resources. These materials include, to a greater or lesser extent, wood–plastic composites, which contain a significant proportion (≈30–70%) [5] of wood (in the form of particles or dust) and may also use a biodegradable matrix or other additives of biological origin. However, their most important contribution to sustainability is the possibility of using recycled materials (plastic, wood, etc.) in their production [4,5,6].

Scientifically based criteria for the strength and fracture resistance of materials are becoming increasingly important in the development of new materials and technologies and in the design and operation of new structures and devices. Regardless of whether well-known materials (steels, metal alloys) or relatively new materials (plastics, composites) are used, different evaluation criteria are applied depending on the type of loading (simple or complex and diverse loading) and the material itself (brittle, plastic) [7]. As the design of increasingly complex engineering structures using composite materials develops, the risks associated with various defects, as well as their propagation and delamination, must be assessed. The most widely used conservative stress/stiffness approach is not sufficient; instead, fracture mechanics criteria must be applied [8]. Although the science of fracture mechanics is relatively new, it has played an important role in the development of safely engineered structures, including those used in transportation (e.g., airframes, gas-turbine engines), construction (e.g., support beams, welded structures) and energy production (e.g., power turbines, pressure vessels and pipelines) [9]. As the science has become more popular, its applications have only increased, and the use of fracture parameters and crack propagation modes has been extended to the analysis and understanding of machining processes for various materials and the causes of tool wear. As a tool penetrates a material and separates and removes it in the form of a chip, different fracture mechanisms occur, the understanding of which helps to improve the composition of the material itself and the design of the tool in order to ensure the longest possible tool life and to obtain the required precision surfaces [10,11]. Material parameters, fracture energy and FZP length are crucial for characterizing and modeling the fracture process and material resistance to crack initiation and propagation [7,12]. Since WPCs contain a significant proportion of wood particles of different sizes, albeit chemically and thermos-mechanically treated, their analysis must look for similarities and differences in crack propagation in the wood itself, since information about the fracture processes, crack propagation modes and parameters of WPCs is still missing. Solid wood fractures occur within a fracture process zone that extends upstream of the crack tip, in contrast to cracked brittle materials, in which the entire fracture process occurs at the crack tip. When the fracture of wood material has begun, additional energy dissipation occurs in the FZP, as compared to a perfectly brittle fracture, resulting in increased energetic resistance to fracture. During the softening of wood, complex fracture mechanisms, such as sliding, friction, decohesion, pulling of bridging fibers out of the gap, as well as anisotropy and material inhomogeneity, are observed. All of these contribute to nonlinear fracture resistance [13]. The fracture strength of wood has been extensively studied, but comparatively little is known about WPCs, which were only developed relatively recently. In WPCs, wood particles can be chemically or physically modified in various ways and with various additives. Their pores are filled with a polymer, so their crack propagation mechanisms and the associated effects are unique compared to solid wood [14]. The overall failure of a new wood–plastic composite, which already has distinctive properties, must be considered separately in addition to the failure of the wood or plastic. The ISO 13586 standard [15] for plastics states that the laws of linear elastic failure mechanics (LEFM) are applied when testing short-fiber (l ≈ 0.1–0.7 mm) polymer composites, which include WPCs, under the three basic conditions of quasi-homogeneity, so that an alignment of the fibers in plain and uniform thickness distribution can be achieved. Otherwise, the laws of non-linear elastic fracture mechanics (EPFM) must be applied. There is still a lack of knowledge about the fracture properties of wood materials and their energy release rate values compared to their strength values, although LEFM laws are included in the Eurocode requirements (EC5) for wood structures and researchers recommend a more comprehensive use of these criteria in design requirements [8]. Due to the wide morphology of material, researchers see challenges in determining such values. Furthermore, a more thorough study of deformation processes requires more sophisticated modeling techniques such as finite element analysis (FEA) [16], which allows for the evaluation of non-linear effects such as friction and shear [8,16].

Today’s globalization of markets and production leads to increasing product complexity and to a growing number of product variants, which is also the case with WPCs. In order to ensure ever shorter product life cycles and meet ever stricter quality standards, highly flexible and reliable production based on advanced manufacturing technologies, digitalization, and automation is used [17]. The use of various simulation technologies and the modeling of complex systems are becoming increasingly important in improving the efficiency of product development and production [14]. The state of scientific development and technological progress means that a variety of methods are now used to study the degradation resistance of materials: these include cohesive zone models (CZMs) with orthotropic continuum models, the finite element method (FEM), digital image correlation (DIC), analysis of displacement and strain fields, acoustic emission, etc. [13]. However, for the application of advanced numerical models to wood and its composites, knowledge of the main failure parameters of the studied material in orthotropic directions is one of the requirements [14], which, in turn, calls for results based on extensive experimental studies. Both wood and its composites are approximated as orthotropic materials [14,16]. When attempting to apply isotropic body assumptions, the results can be subject to large errors and require more complex models [7]. While many researchers are working on developing such complex models, the rupture of WPCs itself involves many complex and interconnected processes that require interdisciplinarity. Therefore, any positive results obtained in the analysis of the fracture strength of WPCs are of great importance for material developers and engineers, as they contribute to the improvement of the materials themselves and to their design, processes and requirements [14].

This study aims to experimentally and numerically investigate the fracture behavior of two different wood–plastic composites in the extrusion directions L and T using SENB tests.

## 2. Materials and Methods

### 2.1. Samples Preparation and Surface Characterization

Two types of solid extruded wood–plastic composites in the form of flat, rectangular decking boards were procured from the local market and used for research: (1) referred to as WPC-A, made from rigid, unplasticized, amorphous recycled thermoplastic polyvinyl chloride (PVC-U) and Scots pine (*Pinus sylvestris*); (2) WPC-B, from the primary processing of semi-crystalline high-density polyethylene (HDPE) and Moso bamboo (*Phyllostachys edulis*). Measured by weight, WPC-A consists of 50% plastic, 45% wood particles and 5% additives, while WPC-B consists of 60% wood particles, 30% plastic and 10% additives. In terms of volume proportion, the WPC-A composite consists of ≈50% wood and 50% plastic, while the WPC-B composite consists of ≈60% wood and 40% plastic.

The plates were processed via CNC turning and milling to produce samples with the required shape, dimensions and orientation. The following abbreviations are used to indicate testing the composite materials in different directions: WPC-A tested in the longitudinal extrusion direction is designated AL, and the same composite tested in the transverse direction is designated AT. WPC-B is correspondingly coded with BL or BT. More specifically, the samples are coded with a number after the letters (AL1, AT2, BL3, etc.) indicating the sample number. The surface of composites was assessed using an Inskam315 LCD optical microscope (Inskam Company Ltd., Shenzhen, China), looking perpendicular to the surface to be examined. For each composite, the diameter and length of 100 visible particles were evaluated, and the average data are presented.

### 2.2. Physical–Mechanical Properties of the Samples

**Figure 1:**
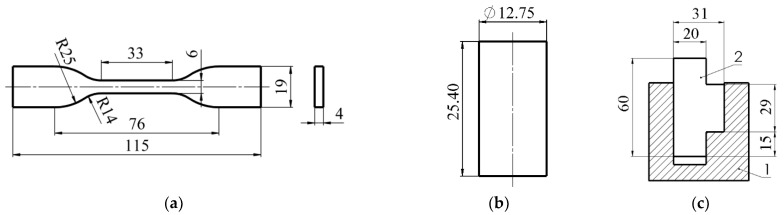
Dimensions of specimens for strength tests: (**a**) tensile strength; (**b**) compression and (**c**) shear: 1—steel holder, 2—WPC.

The main properties of the WPCs (density, hardness, strength) were determined in the laboratory of Vytautas Magnus University. Density was determined by weighing five samples with actual dimensions of Ø 12.75 mm × 25.4 mm and then performing analytical volume and density calculations. Tensile and compression tests were carried out using an Instron 5965 (Instron Corporation, Norwood, MA, USA) universal testing machine, while shear strength tests were carried out using the Toni Technik 2020 press machine (Toni Technik Baustoffprüfsysteme GmbH, Berlin, Germany). The testing speed was 20 mm·min^−1^ (tolerance ± 10%) in all cases. The dimensions of the samples are shown in Figure 1. ASTM D638–14 [18] was used for the tensile test and ASTM D695 [19] for the compression test. One end of the tensile samples was fixed. Shear strength tests were carried out according to the standard LST EN 13354:2009 [20]. A special steel bracket, shown in Figure 1c, was used for the shear strength tests. The thickness of the test specimen for the shear test was 15 mm. The ambient temperature during the tests was 21 ± 1 °C and the relative humidity was 40%. Average data from 3 replicates are shown. Strength values were calculated analytically from the stress–strain curves obtained.

**Figure 2:**
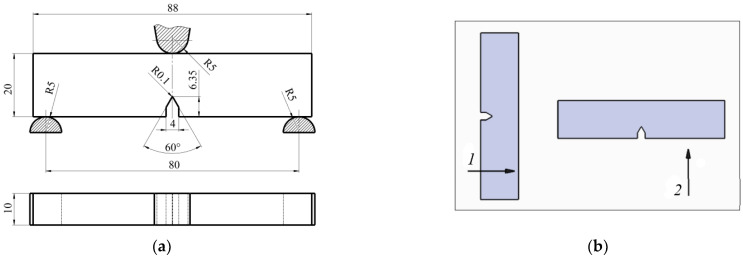
Test specimens for SENB: (**a**) dimensions of the specimen and notch; (**b**) notch orientation when milling the specimens from the plate: 1—longitudinally along the extrusion direction (L), 2—transversally (T).

Figure 2 displays the specifications of the samples that underwent SENB experiments. The tests were conducted in compliance with ISO 13586:2018 (E) [15], and the calculations were also performed in accordance with ASTM D5045–14 [21], which offers examples of calculations and units of measurement. These two standards are complementary to one another. Three samples for testing in both the transverse (T) and longitudinal (L) directions were prepared from each composite. Due to the insufficient thickness of the workpiece (plate), no samples were prepared in the thickness direction (S). Assuming S = T, the wood–plastic composite was modeled as a transversely isotropic material. A granite VHM (Hoffmann Group, München, Germany) solid carbide engraving tool was used to mill the notch (artificial pre-crack).

### 2.3. SENB Calculations

Given that the bend specimen meets the standard condition *S*/*W* = 4 (*S* = 80 mm for the support span and *W* = 20 mm for the specimen width), the critical stress intensity factor can be calculated using the following formula:

(1) $${Κ}_{Q}=\left(\frac{F_{Q}}{BW^{1/2}}\right)f\left(x\right)$$

where *K_Q_* the conditional (trial) value of the critical stress intensity factor *K_Ic_* (fracture toughness), *F_Q_* is the conditional maximum load (calculated using the standard methodology), *B* is the sample thickness, *W* is the sample width and *f* (*x*) is a factor that accounts for the specimen’s geometry and for the shape of the crack. In our situation, the geometry coefficient *f* (*x*) can be calculated according to the following formula:

(2) $$f(x)=6x^{1/2}\frac{\left[1.99-x\left(1-x\right)\left(2.15-3.93x+2.7x\right)\right]}{\left(1+2x\right){\left(1-x\right)}^{3/2}}$$

where *x* = *a*/*W* is initial relative pre-crack length (0 < *x* < 1), and *a* is the pre-crack length (*a* = 6.35 mm) as depicted in Figure 2a.

**Figure 3:**
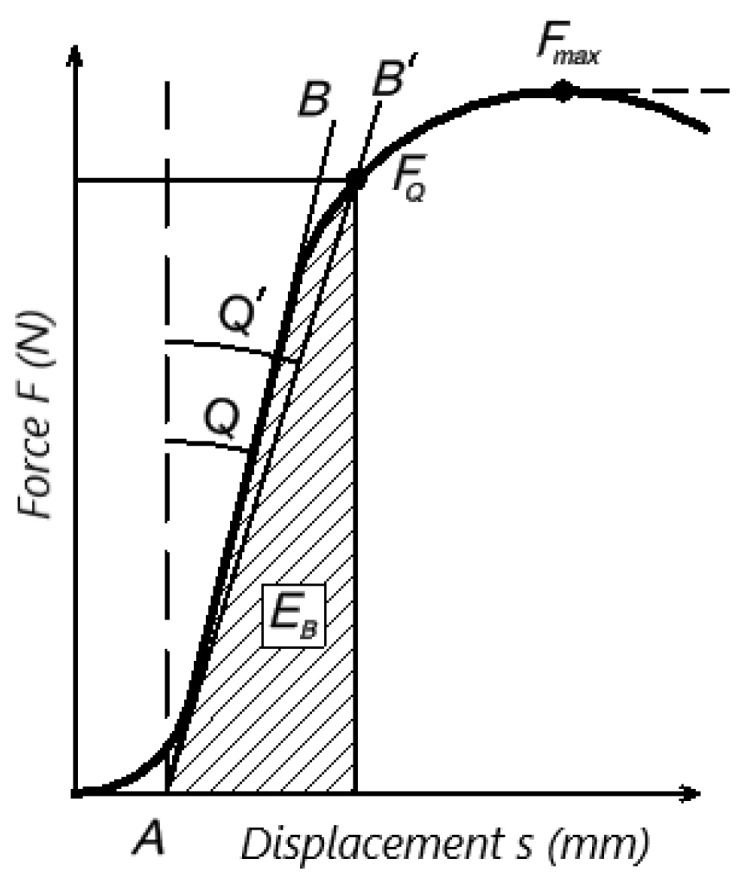
Determination of load *P_Q_*, initial stiffness *S* and energy to break *E_B_*.

The critical strain energy release rate is calculated using the following formula:

(3) $$G_{Q}=\frac{E_{B}}{B\cdot W\cdot \phi \left(x\right)}$$

where *G_Q_* is the conditional critical strain energy release rate (the critical strain energy required for crack growth) of *G_Ic_* value, *E_B_* is the energy to break as shown in Figure 3, *B* is the thickness of the sample, *W* is the width of the sample and *ϕ* (*x*) is the energy factor, which is calculated using the following formula (or taken from the table in the standard):

(4) $$\phi (x)=\frac{A+\;18.64}{dA/dx}$$

where $A=\frac{16x^{2}}{{\left(1-x\right)}^{2}}\left(8.9-33.717x+79.616x^{2}-112.952x^{3}+84.815x^{4}-25.672x^{5}\right)$, $\begin{array}{l} \frac{dA}{dx}=\frac{16x^{2}}{{\left(1-x\right)}^{2}}\left(-33.717+159.232x-338.856x^{2}+339.26x^{3}-128.36x^{4}\right)+\\ \frac{32x}{{\left(1-x\right)}^{3}}\left(8.9-33.717x+79.616x^{2}-112.952x^{3}+84.815x^{4}-25.672x^{5}\right) \end{array}$.

The value of force *F_Q_* to be used in the calculation of *K_Q_* was determined by analyzing the graph of the relationship between force *F* and the opening of the crack (in our case deflection *s*) (see Figure 3). Deflection *s* is also referred to as the load-line displacement (LLD), this abbreviation is also used below. Line *AB*, from which the initial contact stiffness *S_ini_*, N·m^−1^ is estimated and which forms an angle *Q* with the vertical line, is drawn. Then, line *AB^I^* is drawn; it is inclined at 5° and has an angle of *Q^I^* = 1.05*Q*. If the value of maximum force *F_max_* is between lines *AB* and *AB^I^* (inside), the value of *F_max_* is used to calculate *K_Q_*. Otherwise, if *F_max_* is outside lines *AB* and *AB^I^*, the value of *F_Q_* is used to calculate *K_Q_*. In addition, it is a requirement that *F_max_*/*F_Q_* be <1.1; otherwise, the test is rejected if the 10% non-linearity condition is not met.

The condition regarding the geometric dimensions of the workpiece was verified by evaluating elements on the left-hand side of the formula separately:

(5) $$B,\;a,\;(W-a)\;>\;2.5\;{\left(K_{Q}/\sigma_{y}\right)}^{2}$$

where $\sigma_{y}$ is the yield stress to be taken from the maximum load in the uniaxial tensile test. An alternative method is to use 0.7 times the compressive yield stress or the stress at fracture if yielding does not occur and brittle fracture is observed.

When the criteria meet the conditions of the standard, it is considered that *K_Q_* = *K_Ic_*, *G_Q_* = *G_Ic_*.

**Figure 4:**
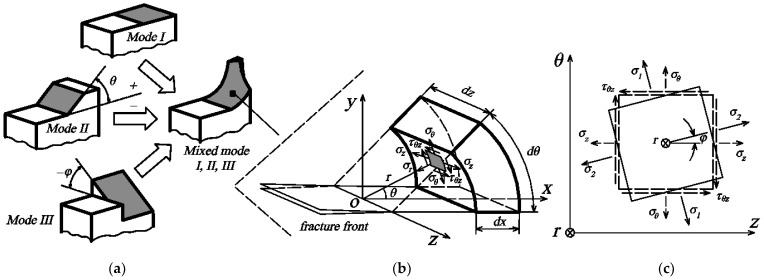
(**a**) Crack propagation surfaces under pure modes and under mixed mode I/II/III conditions; (**b**) stress field components acting on an element near the crack front in polar coordinates; (**c**) stress state in θ-z plane.

Estimation of the mean for the entire study performed with a 95% confidence interval (*CI*) using the formula:

(6) $$CI=\bar{x}\pm 1.96\frac{s}{\sqrt{n}}$$

where $\bar{x}$ is the sample mean, *s* is the sample standard deviation, and *n* is the sample size. The area under all analyzed curves was calculated using the integration tool in OriginPro 2024 (Learning edition, USA). The crack propagation mode was evaluated as depicted in Figure 4a [9,12,22], which illustrates three modes: mode I is the opening mode (a tensile stress normal to the plane of the crack), II is an in-plane shear (a shear stress acting parallel to the plane of the crack and perpendicular to the crack front) and III is out-of-plane shear (a shear stress acting parallel to the plane of crack and parallel to the crack front), as well as using principal and shear stresses acting on the infinitesimal integral element and the kinking angles that affect the direction of crack opening (Figure 4b,c). In practice, composites are characterized by mixed modes I, II and III, but the first mode is the most important and most frequently analyzed [7,9,12].

**Figure 5:**
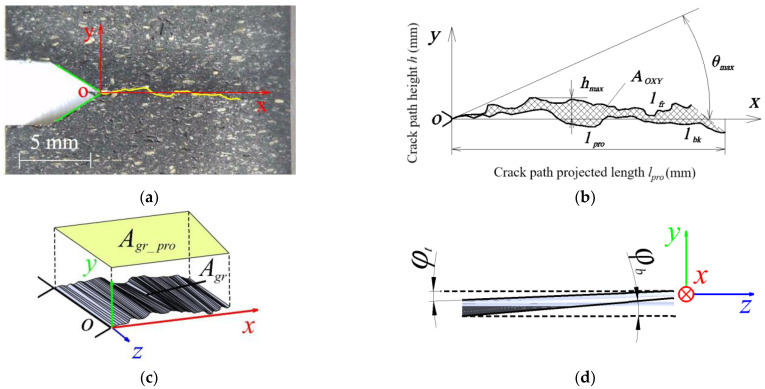
Schematic representation of fracture assessment methods: (**a**) polyline along the crack line; (**b**) anterior and posterior fracture trajectory projection in the OXY plane and evaluation parameters; (**c**) graphical model of the evaluation of the fracture plane and surface; (**d**) graphical representation of the method for evaluating the mean kink angle *φ*.

After the tests, photographs of the samples were taken (at 12× magnification) perpendicular to the front and back surfaces (OXY plane), and a graphical analysis of the main fracture trajectories was carried out. To achieve this, the crack path was traced by a polyline, as shown in Figure 5a, using the nanoCAD 5.0 plotting software. As shown in Figure 5b, the following parameters were also evaluated: mean fracture line length *l_avg_*, mean projected fracture line length *l_pro_avg_*, maximum kink angle *θ_max_* from the origin and maximum fracture height *h_max_* from the OXZ plane. The projected area *A_OXY_* was evaluated as shown in Figure 5c. The following formula was used to obtain the average kink angle φ of the crack plane, as depicted in Figure 5d:

(7) $$\varphi_{avg}=0.5\left(\varphi_{t}+\varphi_{b}\right)$$

Based on the highest *φ_t_* and lowest *φ_b_* fracture surface projection lines in the OXZ plane, the kink angle *φ* was graphically evaluated in Solidworks 2024, as illustrated in Figure 5d.

The average crack length was calculated using the following formula:

(8) $$l_{avg}=0.5\left(l_{fr}+l_{bc}\right)$$

where *l_fr_* is the length of the crack (traced by a polyline) visible on the front surface, and *l_bk_* is the length of the crack visible on the back surface. The projected lengths of *l_fr_* and *l_b_* of each curve on the OX axis were also calculated and averaged. The *l_pro_avg_*/*l_avg_* ratio helps to more accurately assess the curve deflection and identify and characterize the shape of the crack. For an ideal mode I, this ratio is 1, with a higher ratio indicating a more pronounced mode II. Mode II was also more pronounced the higher the value of *h_max_*. The greater the visual difference between the front and back of the curve, i.e., the further apart they are, the more pronounced the shape of mode III and the larger the kink angle *φ* were. A higher *A_OXY_* value will indicate a greater influence of mode III on the effective critical stress intensity factor. In the case of a pure fracture mode I, *A_OXY_* = 0 (*φ* = 0). The area A*_gr_* and the projected area *A_gr_pro_* in the OXZ plane were graphically evaluated by combining the two trajectories *l_fr_* and *l_bc_*, as shown in Figure 5c. The degree of unevenness of the fracture surface can be determined by a higher *A_gr_*/*A_gr_pro_* ratio. Ideally, this ratio should only be equal to 1 in pure mode I. The more uneven the fracture surface is, the more twisted it is, and the higher the area ratio is. These additional parameters developed by the author allow for a better comparison of composites tested under identical conditions and a more accurate determination of the predominant crack propagation mode. Average data are relevant in order to avoid evaluation based only on observed local maxima or minima.

### 2.4. Numerical Modeling

**Figure 6:**
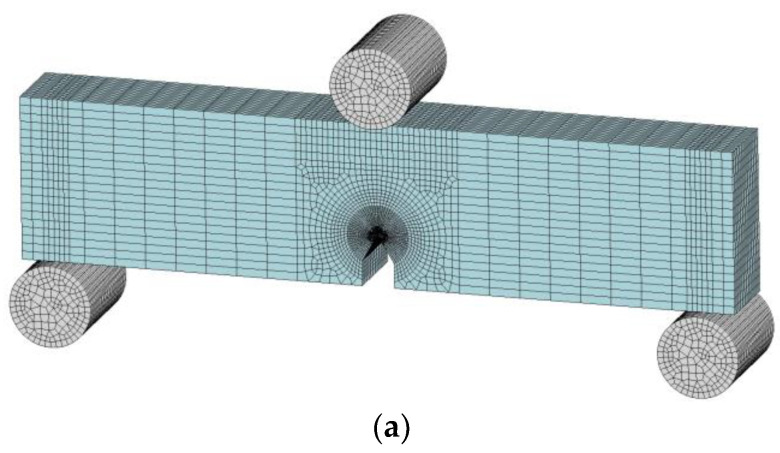

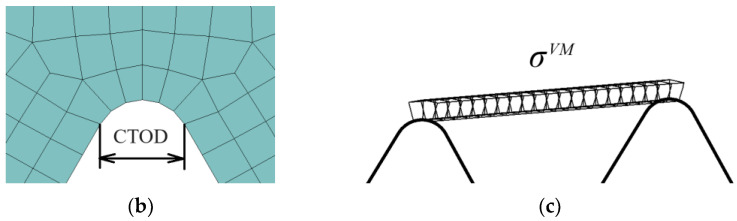
Solid FE model: (**a**) SENB test scheme; (**b**) enlarged view of the vicinity of the crack tip and CTOD measurement site; (**c**) a scheme of the evaluation of the average von Mises stress in the crack front elements.

Numerical modeling was performed in Ansys LS-DYNA Suite R13 Student program, which is limited to a maximum total number of 128 × 10^3^ nodes/elements. The SENB test scheme is shown in Figure 6a. The crack tip opening displacement (CTOD) up to LLD = 0.3 mm (simulation time t = 1.8 s) was evaluated numerically, as shown in Figure 6b. The average absolute difference (CTOD_final_ − CTOD_initial_) over the entire thickness of the sample is also shown (CTOD_initial_ = 0.1732 mm). The CTOD measurement was compared with the vertical load and with the average von Mises stress of the 40 crack front elements, as shown in Figure 6c.

No additional studies were carried out on the deformation of the indenter and supports since they were selected as rigid bodies (MAT_20). The properties of the indenter and supports composed of the same material are listed in Table 1. The values for the stainless steel used to produce these components were taken from a publicly accessible internet source [23]. Our study of these parts did not require precise material properties, as the composites examined were approximately 100 times less hard.

The element type of the MAT_20 solids is a single integration point ELFORM = 1. The indenter consisted of 4508 elements (5220 nodes), and the two supports together consisted of 9016 elements (10,440 nodes). The composite sample was modeled as a full-size deformable 3D solid with element type ELFORM = −2 (8 integration points hexahedral). The selected control type was zero integration energy (hourglass) control type IHQ = 6, coefficient QM = 0.1. The composite consisted of 59,600 elements (64,785 nodes). The total number of elements was 73,124, and the number of nodes was 80,445. The orthotropic wood model MAT_143 was selected, the properties of which were determined in tests and in the result analysis. All directions of movement of the indenter were restricted, except for vertical movement, and the supports were fully restricted in all degrees of freedom. In contrast, the composite required no additional restraint as it was compressed from above by the indenter while resting on both supports from below. By avoiding convergence errors, the unconstrained sample attempted to replicate a real situation as closely as possible. Frictional forces acted between the metal surfaces and the test specimen. The AUTOMATIC_SURFACE_TO_SURFACE_MORTAR contact was selected [24]. Based on tests carried out on an inclined plane, the coefficients of static friction were determined. The resulting inclination angle served as the basis for the formula *tgα* = *μ_s_*_._ The values *μ_s_* = 0.4 (for WPC-A) and *μ_s_* = 0.36 (for WPC-B) were chosen. The sample to be bent was the slave in the MORTAR contact, while the indenter and supports (harder bodies) formed the master body. The correct matching of the MORTAR contact parameters was crucial to obtain the correct reaction forces. In this type of contact, the pressure changes in accordance with the following formula [24]:

(9) $$\sigma_{c}=\alpha \varepsilon K_{s}f\left(\frac{d}{\varepsilon d_{c}}\right)$$

where *σ_c_* is the contact pressure (of the indenter and supports with the composite sample itself), *α* is the scaling parameter for the contact stiffness (SFS × SLSFAC) selected in the program environment, *K_s_* is the stiffness modulus of the slave (softer body) surface segment, *d* is the penetration distance, *ε* is a constant equal to 0.03 and *d_c_* is the characteristic length (length of the smallest finite element edge). The stiffness parameters were tuned based on the initial contact stiffness values obtained by *S_ini_* from the experimental studies.

SENB tests were performed using a load rate of 10 mm·min^−1^ (1.67 × 10^−4^ m·s^−1^), as required by the standards [14,20]. In implicit dynamic analysis (type IMASS = 1), Newmark’s time integration constants (*γ* = 0.6, *β* = 0.38) were utilized to apply minimal dynamic damping to the highest number of converged iterations. Strain rate parameters were not included in the simulation because of the incredibly low strain rate in quasi-static analysis. The elastic moduli (EL, ET), hardening (NPAR, CPAR, NPER, CPER) and softening (BFIT, DFIT) parameters were numerically calibrated with 3-point bending experiment data. Numerical analysis was carried out up to a deflection of 0.3 mm, or approximately 58% of the maximum load, beyond which the solution fails to converge due to intense elemental degradation.

The bodies modeled in the simulation program environment were divided by a finite element mesh. The left and right sides would be identical if the 3D sample model was divided in half through the top of the pre-crack. Given that the radius of the pre-crack tip was *R* = 0.1 mm, the zone was modeled as an arc in the corresponding finite element mesh. Moving from the tip to its sides, the mesh became coarser in less important areas. The mesh was the smallest at the tip of the crack. The size of the smallest element was about 0.05 mm × 0.07 mm × 0.5 mm, and the volume was 1.57 × 10^−3^ mm^3^. The size of the largest elements outside the notch zone was 3.75 mm × 1.0 mm × 0.5 mm.

The equivalent von Mises stresses, stress triaxiality coefficient and Lode angle parameter were all evaluated after completing the numerical experiment. In combination, these three indicators provide an accurate description of the three main scalar invariants of the stress deviator and enable the assessment of the triaxial state of stresses in the deformable material.

The stress triaxiality coefficient (dimensionless) was calculated according to the following formula [7,25,26]:

(10) $$\eta =\frac{\sigma_{m}}{\sigma^{VM}}=\frac{\frac{1}{3}\left(\sigma_{1}+\sigma_{2}+\sigma_{3}\right)}{\sqrt{\frac{1}{2}\left[{\left(\sigma_{1}-\sigma_{2}\right)}^{2}+{\left(\sigma_{2}-\sigma_{3}\right)}^{2}+{\left(\sigma_{3}-\sigma_{1}\right)}^{2}\right]}}=\frac{I_{1}}{3\sigma^{VM}}$$

where *σ_m_* is the average (hydrostatic) stress, which is further expressed by the first invariant of the stress tensor *σ_m_* = *I*_1_/3; *σ^VM^* is the equivalent von Mises stress expressed by the second invariant of the stress tensor *σ^VM^* = (*3I*_2_)^0.5^; and *σ_1_*, *σ_2_* and *σ_3_* are the principal stresses (*σ_1_* > *σ_2_* > *σ_3_*). Coefficient *η* shows the relative size of the hydrostatic and von Mises (yield) stress in a given state of stress [27]. Based on coefficient *η*, the state of stress can be classified into several categories [26]: (1) small *η* (0 < *η* < 0.3) indicates highly constrained fracture zones, with shear loading usually prevailing; (2) average *η* (0 < *η* < 1) is usually characteristic of a crack starting from the free surface; (3) high *η* (1 < *η* < 2), is a classic form of plastic failure in which stress concentration is not high and in which pores or cracks with rounded edges are observed; and (4) very high *η* (2 < *η*) occurs when cracks are observed, usually with sharp edges. There is either a limited yield point or the material does not have it at all, while in the case low of plasticity, traditional fracture mechanics (e.g., J-integral) are perfect. Lode angle parameter *ξ* (−1 < *ξ* < 1) is calculated according to the following formula [27]:

(11) $$\xi =\frac{27I_{3}}{2{\left(\sigma^{VM}\right)}^{3}}=cos(3\theta_{L})$$

where *I*_3_ is the third invariant of the stress tensor, *σ^VM^* is the equivalent von Mises stress and *θ_L_* is the Lode angle (0 < *θ* < *π*/3). Lode angle parameter *ξ* for several typical stress states is as follows: (1) an axially symmetric tensile stress state, where the principal stresses *σ*_2_ are equal *σ*_3_ (*ξ* = 1); (2) an axially symmetric state of compressive stresses, where *σ*_1_ = *σ*_2_ (*ξ* = −1); (3) plain strain, where *σ*_2_ = 0.5 (*σ*_1_ + *σ*_3_) (*ξ* = 0). Setting aside parameter *ξ* on the abscissa axis and coefficient *η* on the ordinate axis, separate quadrants show different states of stress in the material.

## 3. Results

### 3.1. Surface and Structure Description

**Figure 7:**
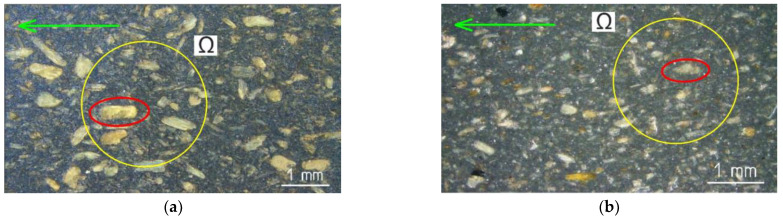
Optical microscope (OM) images of the surfaces of the composites: (**a**) WPC-A; (**b**) WPC-B. The green arrow symbol indicates the direction of extrusion (L-direction), the yellow circle indicates the domain of interest Ω and the red ellipse symbol indicates the measured wood particle.

Images of the two surfaces of composites are presented in Figure 7.

**Figure 8:**
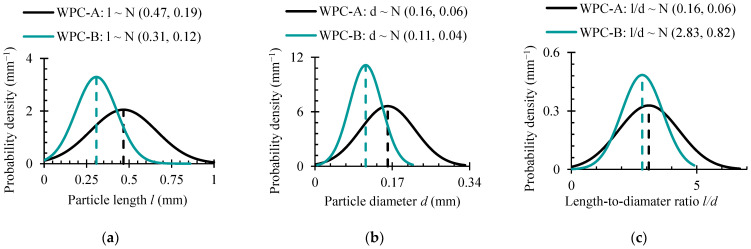
Probability density functions of the normal distribution of the main particle parameters: (**a**) particle length *l*; (**b**) particle diameter *d*; (**c**) particle length-to-diameter ratio *l*/*d*.

The pictures show the shape, size and distribution of particles in the solidified thermoplastic matrix. As can be seen in Figure 7, the WPC-A composite had larger wood particles on its surface, whereas the WPC-B composite had smaller particle sizes, with wood dust being the largest component. The quantification was performed using the optical microscope image. With the observation direction perpendicular to the surface, the length *l* and diameter d of the particles were measured, and the length-to-diameter ratio (*l*/*d*) was calculated. Particle sizes were measured accidentally while investigating multiple domains Ω of different samples. Statistically processed data are shown in Figure 8.

Figure 8 illustrates that in comparison to WPC-B, the average wood particle in the WPC-A composite was approximately 34% longer and 31% larger in diameter. Nonetheless, the flatter distribution curve means that the scatter of WPC-A data was greater. This suggests a higher degree of size uniformity in WPC-B particles within the domain Ω of research. While high scatter in WPC-A makes it challenging to report on meaningful differences in the means, the *l*/*d* ratios of the two composites were comparable, with the WPC-A composite having a slightly higher (8%) ratio.

The shear load transfer from the matrix to the reinforcing particles through the interfacial surfaces is determined by the size and location of the wood particles in the matrix, which, in turn, determines the crack propagation path. Since the fibers in the composites we have studied are short, it is known that the tensile stresses at the ends of the fibers are zero and only increase as the ends approach the particle center longitudinally. In natural fibers, the critical particle length is usually between 0.2 and 3 mm. This means that particles must be longer than this size in order to prevent the fiber from breaking down under tensile load. The particle length-to-diameter ratio, or *l*/*d*, affects the mechanical characteristics of a composite and controls its mechanical response [28]. The crack path lengthens along the wood particle, dissipating energy as it propagates in the AT direction perpendicular to the longitudinal edge of the particle; the particle is stronger than the matrix, with good interfacial adhesion. Figure 7 shows that not all particles were perfectly and equally distributed but were rather at specific angles. Since the crack primarily spreads in the weakest direction between the boundaries separating the wood particles, the distribution of the particles inherently affects its path. It is well known [13] that while stronger wood particles resist the opening of the crack and create what are known as gap-filling bridges on the fiber to slow down crack growth, weaker wood particles are pulled out or broken off. Some wood particles are also pulled apart as the crack opens and the matrix disintegrates. Although these kinds of studies are appropriate for the future, a more in-depth microanalysis of the particles was not the aim of this paper. There is no additional information available from the manufacturer or the literature regarding the particles in the composites that we looked at. Moreover, the preparation, processing or commercialization of the particles in the composites we studied are unknown to us.

### 3.2. Mechanical Tests

**Figure 9:**
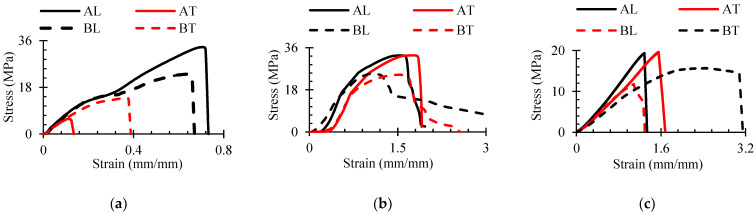
Mean curves of mechanical test results in the L and T directions: (**a**) tension; (**b**) compression; (**c**) shear.

The results of the main mechanical tests are shown in Figure 9.

As can be seen, the tensile strength of composites in the extrusion direction was much higher than that in the transverse direction. In contrast, the compression curves in different directions were very similar, and the maximum strength values of the same composite in the L and T directions were close, with only slight differences. The WPC-A composite exhibited brittle fracture and a steeper plastic failure curve in compression, whereas the WPC-B composite exhibited higher ductility. The shear strengths of the WPC-A composite in the L and T directions were very close. Although the maximum shear strengths of the WPC-B composite were similar, the significantly larger area under the BT curve indicates a higher energy requirement for deformation. The largest differences in properties in the different orthotropic directions were found in tensile strength, with a ratio of AL/AT = 5.2 for WPC-A and a lower ratio of 1.6 for WPC-B. The WPC-B composite also had a larger difference between shear strength values, one of 1.4. The ratio of tensile to compressive strengths is also important [7]. In the L direction, the differences were small, only 1.15 times higher for the WPC-A composite, but in the T direction, they were as much as three times higher for WPC-B than for WPC-A. These differences lead to a complex triaxial stress state during deformation, which is practically impossible to assess without numerical modeling techniques. The results of the analyzed mechanical tests were used for numerical modeling and are presented in Section 3.4.

### 3.3. SENB Experiments

**Figure 10:**
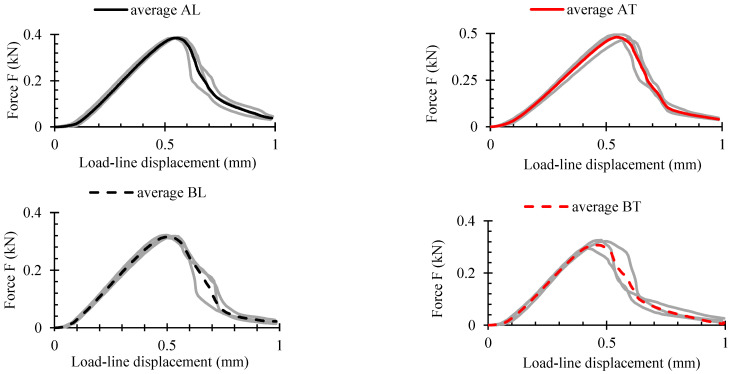

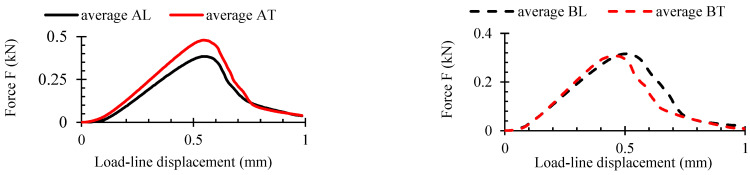
Force-deflection diagrams of SENB specimens.

**Figure 11:**
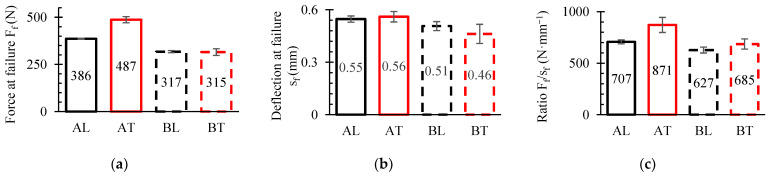
Diagrams of SENB data: (**a**) maximum force (*F_max_*); (**b**) strain to failure (*s_f_*); (**c**) ratio (*F_max_*/*s_f_*).

Figure 10 displays the mean curves (black and red) and the curves of the three tested samples (grey) from the SENB experiment. Figure 11 displays the maximum values attained during the experiment. The mean force-deflection curves were plotted and assessed using OriginPro 2024 (Learning edition, USA). It is evident that up until the maximum load *F_max_* was reached, there was little scatter in the data. However, scatter increased once plastic degradation started, as was also revealed in tensile, compressive and shear tests. The WPC-B composite showed a higher degree of data dispersion, as the SENB curves demonstrated. The dispersion of fracture results was directly impacted by this. In Figure 11, the force magnitude in N per unit of displacement mm is displayed through the calculation of the average relative ratio of maximum force *F_max_* and displacement to plastic failure limit *s_f_* as shown in Figure 11c. As can be observed, more force was needed for crack propagation in the AT and BT directions than in the L direction for both composites. While the differences between BT and BL were smaller, at about 8.5%, the AT direction required an average force per unit displacement that was approximately 19% higher (more work and energy demand) than the AL direction. If the various composites are compared in the same direction of testing, the AT ratio in the T-direction was 21% higher than of BT, and the difference in the L-direction was nearly twice as large. However, the AL composite still had a 10% higher AT ratio than BL.

The results of the SENB parameter calculations are presented in Table 2.

**Figure 12:**
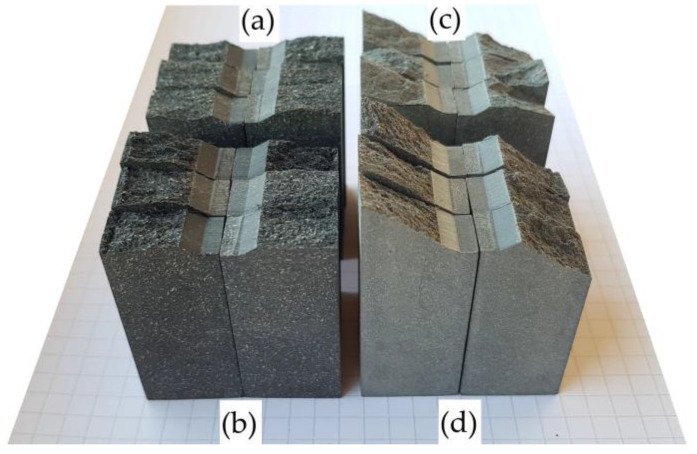
Fractured samples placed on a sheet of paper (grid 5 × 5 mm): (**a**) AL; (**b**) AT; (**c**) BL; (**d**) BT.

**Figure 13:**
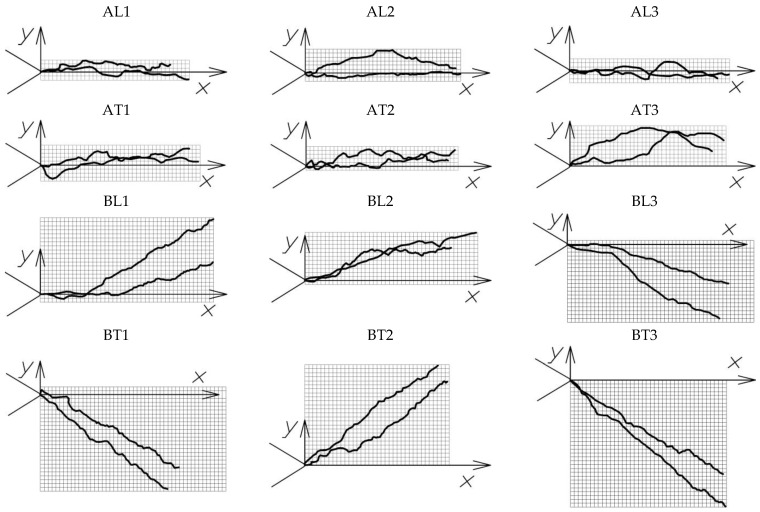
Crack propagation trajectories on the front and rear surfaces (grid size 0.25 × 0.25 mm).

The values obtained are consistent with those reported in the literature for wood and wood composites. In a previous study, the authors indicated that the *K_Ic_* can range from 0.1 to 10 MPa·m^1/2^, while *G_Ic_* can be in the range of 1–20 kJ·m^−2^ [12]. This is quite a wide range. In another work, which studied WPCs using four-point bending [29], although the testing methods and materials are different, the obtained value of *K_Ic_* = 1.79 MPa·m^1/2^ is very close to our results. No other sources were found for comparison, as well as no values for initial contact stiffness *S_ini_*. The non-linearity condition *F_max_*/*F_Q_* < 1.1 was met for all samples, with values in the range of 1.001–1.003. In almost all cases, *F_max_* values were outside the straight lines *AB* and *AB^I^* according to Figure 3 but very close to *F_Q_*. The conditions in Equation (5) were also satisfied. An analysis of the contact stiffness data shows that the initial contact stiffness of the WPC-A composite was higher than that of WPC-B, with a higher resistance to elastic deformation in contact with the indenter. The highest stiffness values were obtained for the orientation of the crack along the transverse direction T of the composite, as the expansion of the crack under tensile forces was hindered by the higher tensile strength of the composites in the longitudinal direction L. The stiffness of WPC-A was 15.5% higher than that of WPC-B when the crack was oriented in the L-direction, whereas for the T-direction, this ratio was very close to 16%. Comparing the *S_ini_* values of the same composites in the L and T directions, the difference between the values in the L and T directions was similar for both composites and was about 13%. These data were used to calibrate the contact parameters during the development of the numerical model. Images of all four sets of fractured samples are presented in Figure 12. The crack paths on the front and rear surfaces in the OXY projection plane are shown in Figure 13, and the crack path calculation data are given in Table 3.

As can be seen in Figure 13, the crack can propagate in either the straight or the negative direction to the *Y*-axis. In the AT direction, compared to the AL direction, the kinking of the fracture surface was about 40% higher, on average; the maximum point of the curve was also higher from the OX axis and the crack path was slightly longer, suggesting a more pronounced mode II crack propagation pattern. The mode III form was similar in both cases but was unstable due to the large errors observed. In all WPC-A tests, the maximum kink was observed at the very beginning of crack propagation. Thus, in the BT direction, compared to BL, the fracture surface, on average, kinked more, even up to several times more. The crack usually had the highest kink angle *θ* (about the OZ axis) in the BL direction, but there was a less frequent case where the kink angle at the beginning of the crack was small, up to 4°, but the highest kinking was observed along the longer section of the path. In the BT direction, the maximum angle was observed at the very beginning in all cases. Even up to several times more pronounced were WPC-B modes II and III. Overall, the probability of the crack starting to kink to one side or the other at the very beginning of the crack opening was >80%.

**Figure 14:**
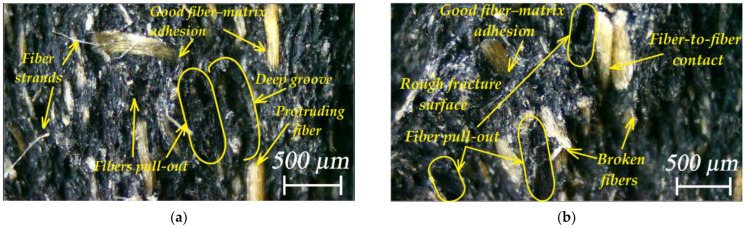

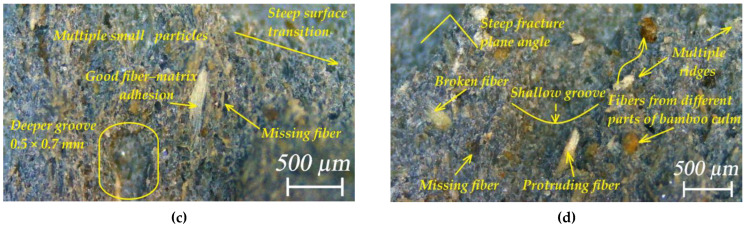
Optical images of fracture surfaces: (**a**) AL; (**b**) AT; (**c**) BL; (**d**) BT. The crack propagation direction was vertical and upward.

Figure 14 shows images of the fracture surface of WPC-A and WPC-B composite specimens after testing. It can be seen that the surface of WPC-A was significantly rougher than that of WPC-B. Due to the coarser wood particles in WPC-A, its higher resistance to deformation and more pronounced decomposition phenomena, such as fiber pull-out, breakage, residues of decomposition products, etc., were more evident. For the deformation of coarser particles, the amount of energy required to break or tear them out with good adhesion to the matrix was higher. In contrast, the fracture surface of the WPC-B composite appeared smoother over a small area, with no major indentations left by the tearing of smaller particles from the matrix. Nevertheless, the surface showed variously shaped indentations with larger blunt edges, torn out particles, ridges of plastic with wood dust formed during deformation and steeper transitions in the fracture planes. The fracture surfaces were also strongly influenced by the material of the matrix, with the WPC-A matrix being largely amorphous, whereas WPC-B was semi-crystalline.

### 3.4. Numerical Modeling

Based on the results of the mechanical tests, the data obtained were analyzed and are presented in Table 4, where the abbreviations of the indicators correspond to the Ansys LS-DYNA Suite R13 Student dialogue window. Several free online user guides can be referred to for a more detailed evaluation [16,30]. Due to their relatively large volume, the equations describing the material model are not presented in this work. This material model was further used to model the WPCs. The MAT_143 wood model [22] assumes that the composites under study included a sufficiently high content of wood particles (50–60%) to allow for the modeling of the variations in their properties depending on the direction. Also, in material property tests, the deformation curves of the model showed significant similarities with the curves of pure wood in terms of both tension and compression. MAT_143 is an elastoplastic damage model, which uses separate elliptical yield surfaces to model longitudinal and scalar damage. Scalar damage formulations account for the progressive reduction in tensile and shear stress components while maintaining ideal plasticity in compression. The hardening of the material was modeled by varying the position of the yield surface, thus evaluating the non-linear response of the material in compression. The wood model itself was developed in 2002, and the first user guide for the wood model MAT 143 was implemented in LS-DYNA in 2007 [16].

The exact orientation and micro properties of the wood particles were not analyzed in this work, as the main material property tests were carried out at the macro level. During composite manufacturing by extrusion, wood particles are oriented in the extruder in the direction of molten plastic flow, which is also the orientation of composite’s molecule chains in general [31]. The composite boards from which the samples were prepared were rectangular, much longer than they were wide or thick, and their relatively simple geometry resulted in a more even and more linear distribution of wood particles. The length of the particle itself was greater than its diameter, which resulted in different properties of the composite in the longitudinal and transverse directions.

**Figure 15:**
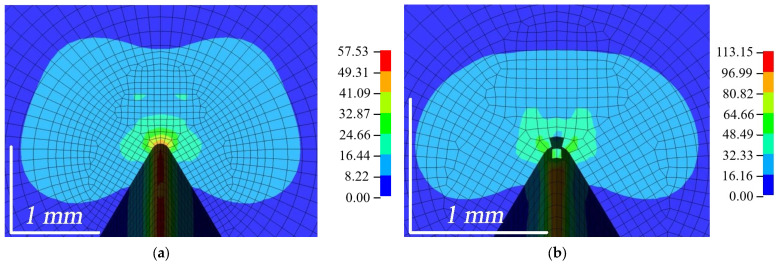

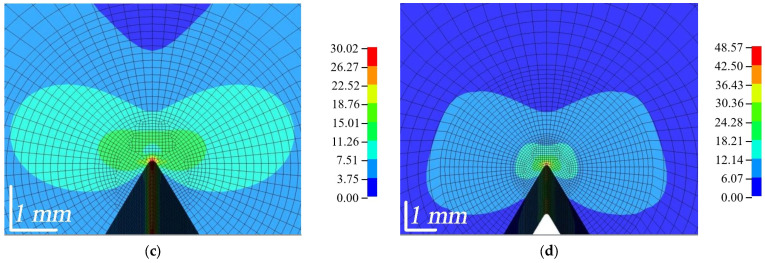
Distribution of equivalent von Mises stresses *σ^VM^* (in MPa units) at LLD = 0.3 mm: (**a**) AL; (**b**) AT; (**c**) BL; (**d**) BT.

The distribution of the equivalent von Mises stresses is shown in Figure 15. It was observed that the failure of elements started at the top of the outer surfaces, while the plastic zone was more constrained in the inner part of the composite [7]. The highest stresses were obtained when bending the WPC-A composite specimen. As can be seen in Figure 15b, once the AT composite started to fail, the stresses around it reached ≈50 MPa. The stresses were higher in the unbroken zone of the crack front, which was more constrained. The AL composite was very close to the limit of failure, with stresses up to 50 MPa. In contrast, the composite in the BL direction had the lowest stresses, around 30 MPa, while BT was also close to the limit of failure with stresses close to 50 MPa at the crack tip. The plastic zone of WPC-B was more pronounced, indicating a higher plasticity of this composite.

**Figure 16:**
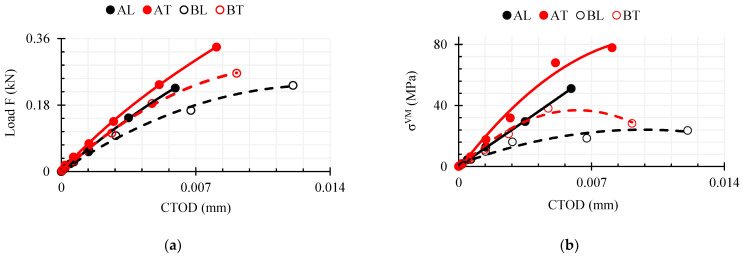
(**a**) Plot of CTOD versus vertical load; (**b**) plot of CTOD versus average von Mises stresses of crack apex elements.

Figure 16 displays the dependencies and CTOD distance numerical estimation data at time t = 1.8 s (LLD = 0.3 mm) based on Figure 6.

**Figure 17:**
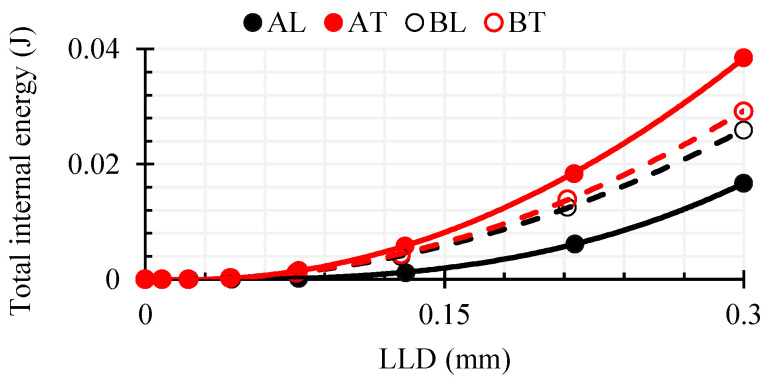
Variation of the total internal energy of a composite specimen with vertical displacement of the indenter.

As can be seen in Figure 16a, with both composites deforming in the transverse direction T and with the same vertical deflection LLD = 0.3 mm, a higher force was required than in the L direction. The maximum force in the AT direction at this loading time was about 1.48 times higher than that in AL, although in AT, the width of the crack tip CTOD was 1.36 times higher. Meanwhile, the difference in the magnitude of the maximum force between BT and BL was small, only 1.14 times. In contrast to WPC-A, the width of the crack apex was 1.32 times larger in the BL direction than in BT. The area under the curve was 1.2 times larger in BL, indicating that the composite had a higher ductility in this direction. In contrast to WPC-B, the area under the curve was about 2.1 times larger in the AT direction, which means that the energy demand for deformation in that direction was twice as large as in AL. As can be seen in Figure 16b, the mean AT von Mises stress *σ^VM^* at the top of the crack reached a relatively high value and the elements started to erode. In contrast, in BT and BL, the stresses were in the range of 30–40 MPa and did not tend to increase further; only the CTOD distance increased, and the composite was characterized by more plastic degradation. The total internal energy of the composite specimen (excluding the energy of the eroded elements) was evaluated from the LD-DYNA database MATSUM and is shown in Figure 17. Internal energy includes the elastic strain energy and the work needed to deform the material plastically. It is calculated based on tensorial values (which in turn are based on six stress and strain components) [32]. The results of numerical simulations show that at a strain of LLD = 0.3 mm, the only elements of the AT composite that had started to erode were the AT elements, but their energy was only 0.02% of the total internal energy in direction AT.

**Figure 18:**
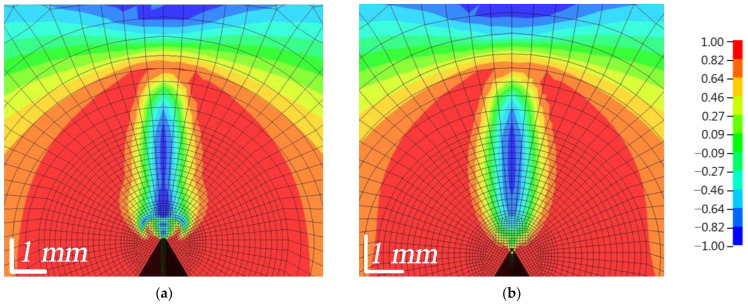

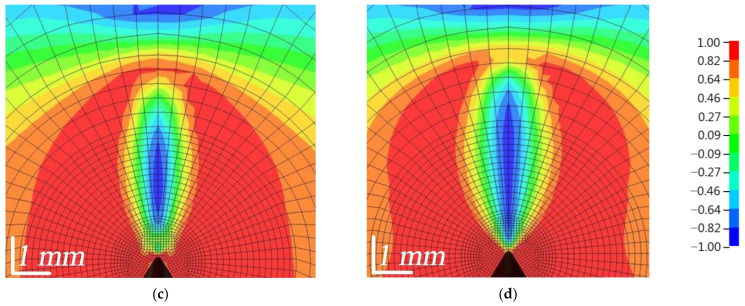
Distribution of Lode angle parameter *ξ* within the composites around the crack tip at LLD = 0.3 mm: (**a**) AL; (**b**) AT; (**c**) BL; (**d**) BT.

The distribution of the Lode angle parameter *ξ* is shown in Figure 18. In the red zone, where the value of the parameter is equal to 1, pure tensile stresses predominated, while in the blue zone, where the value is equal to −1, pure compressive stresses occurred. In the FZP zone, a zone of flame-like stresses was formed; compressive stresses predominated in the central part, surrounded by a narrower zone of shear stresses and finally a large zone of tensile stresses. As the load was increased, the shape of the flame-like stress zone changed, and as the composites began to fail, the bottom of the zone near the top of the crack became uneven, jagged and jellyfish-like. As can be seen, WPC-A started to erode in the AT direction and soon started to erode in the AL direction, as indicated by the von Mises stresses, while WPC-B was still in the process of failure. Shear stresses in the FZP zone had a significant influence on the opening of the crack. The concentration of the wood particles, their size, orientation and the strength of adhesion to the matrix had a major influence on the opening of the crack and its growth.

**Figure 19:**
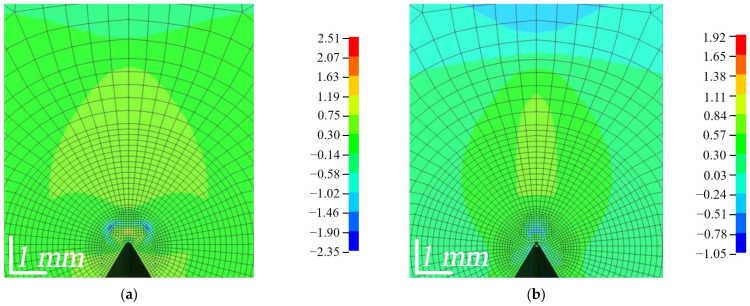

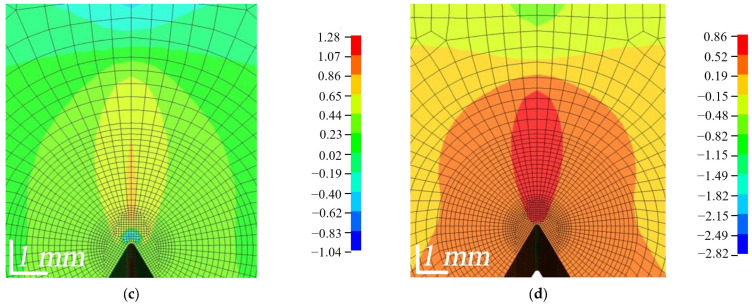
Distribution of stress triaxiality coefficient *η* within the composites around the crack tip at LLD = 0.3 mm: (**a**) AL; (**b**) AT; (**c**) BL; (**d**) BT.

The distribution of stress triaxiality within the composites is shown in Figure 19. The stress sign was positive for tensile stresses and negative for compressive stresses. It can be seen that the fracture process in the FZP was constrained, with a low value of *η*, around 0.4, indicating a strong influence of shear stresses. However, as the crack opened, ductility in the FZP zone increased. As can be seen, the WPC-B composite was characterized by a more ductile fracture, with the narrow red BT zone showing a value close to 1, indicating a pronounced zone of tensile stresses, while the surrounding area was dominated by an orange zone with a coefficient value close to 0, indicating a large zone of high shear stresses. As can be seen in Figure 9c, the average area of BT under the shear curve was relatively large, so a larger amount of energy was required in the BT direction, as reflected in Figure 19d.

### 3.5. Validation of Modeling Results

**Figure 20:**
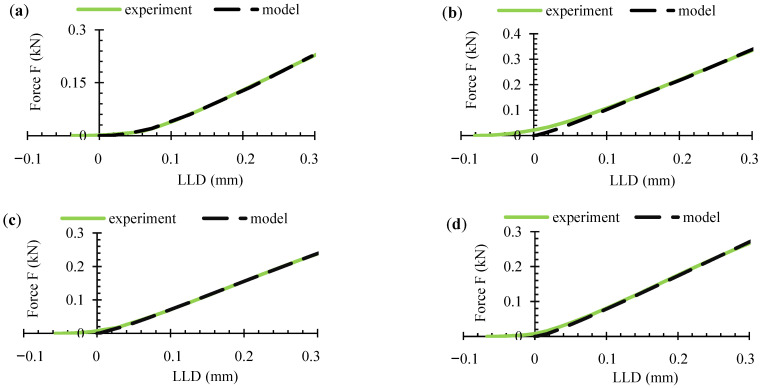
Comparison of experimental and simulation force-deflection curves: (**a**) AL; (**b**) AT; (**c**) BL; (**d**) BT.

**Figure 21:**
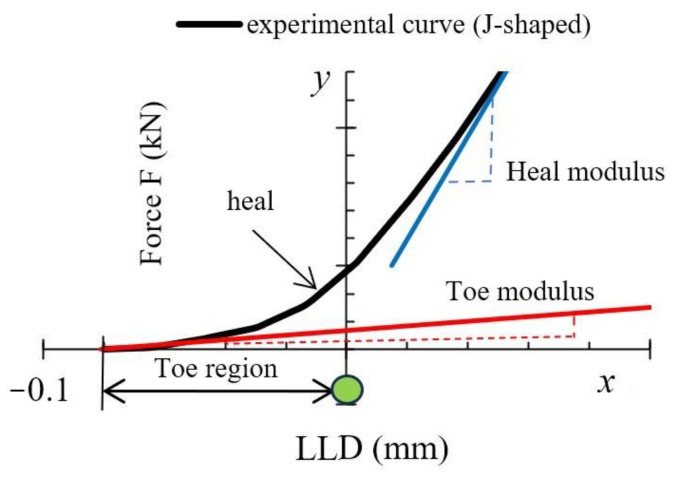
Graphical explanation of toe compensation and evaluation methodology.

The curves from the numerical simulations were compared with the average experimental curves, and the plots are shown in Figure 20. The experimental curves can be seen to have shifted along the *x*-axis and therefore start from a certain negative number. A graph is provided accordingly to show the compensation made. Figure 21 provides a more detailed illustration.

Toe compensation is required by the standards [15,18,21], since it is assumed that the non-linear portion is connected to slack, measurement errors and other factors. The following toe compensations of the experimental curve were made in this work (in mm): 0.037 (AL), 0.081 (AT), 0.057 (BL), and 0.067 (BT). This toe compensation also occurred in the *x*-axis. As seen in Figure 20, the curves were shifted rather than being displayed from the *x*-axis zero point. This was done to draw attention to the fact that others studying WPCs may run into similar circumstances. Not all authors agree on the need for curve shifting and to what extent it is related to the measurement errors of the instrument itself and the processes occurring in the material; therefore, this compensation has been made based on both experimental and numerical data. The initial part of the deformation curves of the materials resembles a J shape, which is a widespread deformation behavior, as it is characteristic of biological soft tissues (skin, arterial walls, collagen), as well as textiles, etc. This deformation curve is also very important for the analysis of polymer composites. The J-shaped curve is divided into three regions [31,33]:

1. The initial region of the stress-strain response, i.e., phase I (toe region).
2. Phase II (heel region).
3. Phase III (linear region).

In the first phase, the molecules of the material were unstressed and branched, so that even a very small stress would cause a sufficiently large deformation without stretching the plastic molecules or fiber. This region was almost linear in practice and has a very low elastic modulus (toe modulus). Materials may have high ductility, although *K_IC_* may be very low, but due to the very low shear modulus in this zone, the released strain energy of deformation cannot be transferred to the FPZ [33]. In the second phase, the material exhibited a non-linear mechanical behavior. The molecular chains straightened and lengthened with load. At the same time, the wood fibers in the matrix aligned themselves in the direction of the load, which results in a higher carrying load. Since the area under the curve in the second zone was small compared to that in the third zone, the amount of deformation energy released was also small. Finally, in the third phase, the material became more rigid, and a linear part was observed as the molecules aligned in the loading direction. The length of the first zone may have been large enough for tissues of biological origin, in the case of this study. Comparing the amount of curve shift in the *x*-axis with the maximum shift before composite failure (≈0.99 mm), the shift values (%) were as follows: 3.74 (AL), 8.18 (AT), 5.76 (BL), 6.77 (BT). It can be seen that a higher discrepancy and nonlinearity were obtained when the composite was deformed in the T direction. Nevertheless, the discrepancies were small compared to the overall result. Although the MAT_143 model produced a non-linear part of the curve at the initial time point, it was not quite as close to ideal bending as the experimental one. The numerical model itself, while performing well, does not contain more detailed parameters describing viscoelastic behavior to more accurately model the behavior of the material in the first phase, when the molecules have only just experienced load and when strains were large compared to the tiny stresses, which means the simulation result is less accurate in representing the processes occurring in this complex initial phase.

The analysis of the curves showed that cubic (third-degree polynomial) equations, rather than exponential equations, are the best descriptors of non-linear behavior, so they were chosen to minimize errors in the comparison (Table 5). The comparison of the two curves (experimental and numerical) was carried out from *x* = 0 (the origin of the coordinates) using the mean absolute percentage error (MAPE), which was calculated according to the following formula:

(12) $$\mathrm{MAPE}=\frac{100\%}{n}\cdot \sum_{i=1}^{n}\left(\frac{\left|E_{i}-M_{i}\right|}{E_{i}}\right)$$

where *n* is the number of data points, *i* is the index of summation, ∑ is the summation notation, *E* is the experiment value, and *M* is the modeling value. The comparison data are shown in Table 5. The calculated MAPE values vary between ≈10 and 15%, indicating small percentage differences between simulated and experimental results. A high accuracy is considered to be achieved when MAPE < 10, so the values obtained in this study indicate a good level of prediction (10 < MAPE < 20). Since the numerical model is quite close to the experimental data, it can be concluded that the distribution of the stresses inside the specimen obtained from the numerical simulation is also sufficiently accurate and able to reflect a realistic situation during the deformation of the WPCs.

## 4. Discussion

Two wood-plastic composites were investigated in the work, which differ both in plastic (amorphous rigid PVC-U and semi-crystalline HDPE), in the type of filler (pine particles and bamboo dust) and in the amount of additives. One composite was 50% wood and 50% plastic by volume, while the other was 60% and 40%, respectively.

It is not possible to numerically model wood dust and particles in a study like this one, as the finest wood dust particles are the size of single microns. In reality, wood dust is not ideally oriented in the extrusion direction of the plastic matrix; it also has a greater or lesser adhesion to plastic, a wide range of fractions and properties and a significant impact on the degradation process. Thus, the numerical model we used was of a load-simulating nature and can be used for analyzing the applied loads, stresses and strains and for investigating the cause of failure. This study used an implicit modeling approach, which is more suitable for static or quasi-static analysis at low strain rates. The solution did not converge once the material started to erode, and other modeling approaches based on non-continuum mechanics are needed, for example, in cases in which a crack could propagate through the element itself but not between the elements. In the future, other simulation techniques, such as the extended finite element method (XFEM), the virtual crack closure technique (VCCT) or other simulation programs intended for a more detailed analysis of cracks, e.g., ABAQUS, could be applied. These methods, which should be suitable not only for shell elements but also for 3D solid orthotropic composites, have not yet been implemented in the LS-DYNA program.

Our experiments were carried out up to full failure of the workpiece and numerical simulations up to a deflection of 0.3 mm, or ≈ 58% of the maximum load. Simulations up to the first cracks are considered to be the most important because once the maximum load is reached, the subsequent failure process develops quite rapidly. The failure of the elements (micro-cracking) started as early as at 50–60% of the maximum load, depending on the quality of the composite, its porosity, etc. The subsequent solution did not converge to this implicit method for intensive element degradation, so the element removal function MAT_ ADD_ERROSION, although attempted, was not applied in the final version. Fracture and damage modeling with element erosion can also be carried out using other methods and programs by selecting different material models with a larger number of parameters, requiring a larger number of tests. The MORTAR contact was the only type that has been converged with WPCs during implicit simulation, and there are no known applications of this contact to date, either MAT_143 material or to WPCs. With materials of varying stiffness (car-to-human dummy contact, steel-to-rubber, heart vessel-to-catheter, etc.), the MORTAR contact is frequently used to solve complex contact problems, where it is challenging to reach convergence [24]. MORTAR is thus suitable for investigating our WPCs‘ contact with stainless steel. The MAT_143 model has been applied to many wood species and composites (balsa, pine, spruce, birch, hardwood, laminated wood [34], etc.), but the amount of research is relatively small, and practical application examples are lacking. MAT_143 itself includes many criteria, but it is not fully developed in terms of specific subtleties, as wood itself is a complex anisotropic material and a large amount of research is required, including not only the determination of material properties but also programming.

The requirements of the standards [15,21] used in this work were developed for isotropic or near-isotropic materials according to defined criteria, which our materials have met. Otherwise, the laws and methods of nonlinear fracture mechanics, of which the J-integral method is the most widely used, must be applied. Although the Ansys LS-DYNA Suite R13 Student software has a tool for the evaluation of the J-integral, it has not been applied in this work. Nevertheless, this paper contributes to a better understanding of the behavior of this group of engineering composites, to a more accurate assessment of uncertainties in loading during operation and to the development of a more accurate methodology for the evaluation of orthotropic composites. On the other hand, based on the standards [15,21] and for future specimen preparation, the *W*/*B* ratio could be increased to 3 or even 4, as is possible in the standards (in the present experiment, it was 2). The notch could be lengthened slightly to 8–10 mm to give a larger difference between the left and right sides of Equation (5), which is desirable under the conditions of the standard. This would perhaps further reduce the influence of ductility and non-linearity when testing WPC composites with bimodal properties. This is the first time that a study of this kind has been carried out without prior knowledge of all the possible nuances.

The causes of the kinking of the crack propagation path are still not fully understood [14,35] and scientifically validated. The mathematical calculations developed to explain them are quite complex [35]. Various theories have been put forward and there are conflicting discussions among scientists, it has been observed that not everything can be explained by the variation in the stress field alone and new methods of verification have been proposed [14]. To create materials with a controlled crack path, scientists are motivated to conduct more thorough research as a result of this. This is particularly important for critical structures in aviation, space and anywhere where major catastrophes and human and material losses could occur.

A wide range of parameters were used to analyze the fracture process following the completion of the experiments in order to more precisely determine the intricate process of crack formation and propagation in this kind of composite material. During numerical modeling, it was not possible to only apply the von Mises criterion, because it describes solely the second invariant of the stress deviator. Therefore, additional parameters were used for the evaluation of material failure in practice: the stress triaxiality coefficient (*η*) and the Lode angle parameter (*ξ*). These are applied even in cases where conventional methods (stress intensity or J-integral) cannot be applied, and they are widely used in computer simulation programs, including numerical models of many materials [25,30]. In fact, in the case of a complex triaxial state of deformations, the plastic failure parameter *ε_pf_* is a function whose variables are not only *η* and *ξ* but also *ė_p_* (rate of plastic deformation), *T* (temperature) and *l_o_* (finite element size) [30]. In this paper, the influence of only a few parameters was investigated.

In the future, it would be appropriate to study the fracture parameters of these composites at different ambient temperatures and various deformation rates (performing not only static but also dynamic tests) by changing the concentration, size, and type of wood particles, modifying the method of wood fiber processing (chemical, physical) and conducting tests with a larger number of specimens. It would also be relevant to determine failure modes II and III by performing additional tests according to other standards. CMOD studies should be carried out by measuring crack opening with special strain gauges and the results should be compared according to the LLD method. Additionally, it would be appropriate to use the DIC method, and other available equipment, as well as to select and test other numerical models of composite materials.

The goal of this study was not a detailed mathematical analysis or the application of one theory of 3D crack formation or another, although many criteria (stresses, deformations, energy) for the evaluation of mixed forms of crack propagation modes I, II and III have been proposed [21]. Instead, our work intends to reveal and highlight essential points, with the possibility of wider research in the future. Only by knowing the properties of a material’s failure can one begin to think about its potential uses in real working conditions.

## 5. Conclusions

This study examined two different wood-plastic composites (WPCs) in two orthotropic directions—in the longitudinal direction L, which coincides with the direction of extrusion of the composite and the direction T perpendicular to it. Fracture properties and parameters of the composites were determined via SENB tests carried out at a constant ambient temperature of 21 ± 1 °C and relative humidity of 40% until the samples failed. Computer simulation using the finite element method and implicit time integration scheme was performed to 58% of the maximum load. By analyzing the mechanical test results and using the orthotropic wood model MAT_143, a numerical model was developed that can be used for future studies on wood-plastic composites. The main conclusions are as follows:

1. When testing to maximum load, the WPC-A composite required, on average, ≈0.12 J of energy for crack opening in the transverse T direction, and it was 1.23 times more than in the longitudinal direction L (≈0.10 J); with the WPC-B composite, this ratio was much smaller, reaching 1.17 times (0.07 J for T direction and 0.06 J for L direction, respectively). After the maximum load was reached, micro-cracks and element failure started. The mechanical characteristics of the composites, which are governed by wood particle size, distribution and strength of adherence to the matrix, had an impact on the variations in the results.
2. Mixed failure modes characterize WPCs. When a crack first opens, it typically kinks at an angle of 30–70° perpendicular to the crack front (strong mode II), while the kink angle parallel to the crack front is much smaller, reaching only 5–7° (weak mode III). Failure mode I is more prominent in composite WPC-A compared to WPC-B. The smaller wood particle size and semi-crystalline matrix structure in WPC-B are strongly correlated with a smoother fracture surface.
3. FEA and experimental results showed a good percentage agreement (MAPE was in the range of ≈10–15%). This demonstrates the applicability of the MORTAR contact and the MAT_143 wood model for implicitly modeling WPCs before the onset of the first crack. The failure process was determined by the formation of a complex triaxial stress state during deformation, when high equivalent stresses prevailed at the edges of the crack tip, and shear stresses in the FZP had a significant influence on the crack propagation process.
4. The force-displacement curves of the SENB test up to the moment of the onset of cracking were characterized by a J shape, which revealed the complex internal processes taking place in the composite during its deformation. Experimental and numerical curves were most accurately approximated by cubic equations (*R^2^* > 0.99).

## Figures and Tables

**Table 1:** Mechanical properties of the indenter and supports of the MAT_20 model.

Material	Density [kg·m^−3^]	Young’s Modulus [GPa]	Poisson’s Ratio
AISI A2 tool steel	7860	200	0.28

**Table 2:** Main parameters obtained in the SENB test.

Parameter	WPC-A		WPC-B	
	AL	AT	BL	BT
*K_Ic_*, [MPa·m^1/2^]	1.74 ± 0.01	2.19 ± 0.07	1.42 ± 0.02	1.42 ± 0.08
*G_Ic_*, [kJ·m^−2^]	1.24 ± 0.04	1.67 ± 0.04	0.94 ± 0.04	0.87 ± 0.17
*S_ini_*, [N·m^−2^]	0.97 ± 0.03	1.13 ± 0.09	0.82 ± 0.04	0.94 ± 0.08

Source: own elaboration.

**Table 3:** Average evaluation parameters of crack propagation path and fracture surfaces.

Parameter/Ratio	AL	AT	BL	BT
*l_avg_* [mm]	10.03 ± 0.60	10.62 ± 0.12	11.51 ± 0.65	11.86 ± 0.96
*l_pro___avg_* [mm]	9.28 ± 0.63	9.41 ± 0.49	10.21 ± 0.52	8.95 ± 0.70
*l_avg_*/*l_pro___avg_*	1.08 ± 0.01	1.13 ± 0.05	1.13 ± 0.01	1.33 ± 0.00
*h_max_* [mm]	0.91 ± 0.52	1.49 ± 0.87	4.13 ± 1.13	6.85 ± 1.35
*θ_max_* [°]	38.79 ± 21.01	61.33 ± 6.13	31.84 ± 11.49	74.08 ± 19.39
*A_OXY_* [mm^−2^]	4.98 ± 3.23	5.45 ± 2.092	10.42 ± 6.53	10.51 ± 0.16
*A_gr_* [mm^−2^]	97.90 ± 6.09	102.04 ± 1.25	113.28 ± 6.84	115.12 ± 9.69
*A_gr_pro_* [mm^−2^]	92.70 ± 6.23	93.07 ± 2.95	102.18 ± 5.02	89.35 ± 6.70
*A_gr_*/*A_gr_pro_*	1.06 ± 0.01	1.10 ± 0.02	1.11 ± 0.01	1.29 ± 0.01
*φ_avg_* [°]	4.93 ± 1.78	4.62 ± 1.69	6.58 ± 3.19	4.73 ± 1.85

**Table 4:** Basic parameters of the transversely isotropic wood material model MAT_143.

Character	Name	WPC-A	WPC-B
RO	Mass density [kg·m^−3^]	1380	1324
EL	Parallel normal modulus [GPa]	3.48	2.62
ET	Perpendicular normal modulus [GPa]	3.38	2.61
GLT	Parallel shear modulus (GLT = GLR) [GPa]	2.04	1.78
GTR	Perpendicular shear modulus [GPa]	1.34	1.55
PR	Parallel major Poisson’s ratio	0.33	0.35
XT	Parallel tensile strength [MPa]	34.19	23.17
XC	Parallel compressive strength [MPa]	32.70	25.51
YT	Perpendicular tensile strength [MPa]	6.56	14.70
YC	Perpendicular compressive strength [MPa]	32.71	24.44
SXY	Parallel shear strength [MPa]	20.15	12.34
SYZ	Perpendicular shear strength [MPa]	20.17	17.11
GF1‖	Parallel fracture energy in tension [J·m^−2^]	543.62	513.80
GF2‖	Parallel fracture energy in shear [J·m^−2^]	376.76	571.23
BFIT	Parallel softening parameter [%]	20	20
DMAX‖	Parallel maximum damage [%/100]	0.9999	0.9999
GF1P	Perpendicular fracture energy in tension [J·m^−2^]	84.58	168.50
GF2P	Perpendicular fracture energy in shear [J·m^−2^]	1006.22	754.62
DFIT	Perpendicular softening parameter [%]	20	20
DMAXP	Perpendicular maximum damage [%/100]	0.99	0.99
NPAR	Parallel hardening initiation [%/100]	0.49	0.28
CPAR	Parallel hardening rate	1100	1736
NPER	Perpendicular hardening initiation [%/100]	0.5	0.28
CPER	Perpendicular hardening rate	280	434

Source: own elaboration.

**Table 5:** Functional expressions for the mean experimental and simulation curves.

Code	Experiment Equation	*R* ^2^	Model Equation	*R* ^2^	MAPE
AL	y = −5.378x^3^ + 3.939x^2^ + 0.070x − 0.001	0.99	y = −5.969x^3^ + 4.133x^2^ + 0.061x − 0.002	0.99	14.57
AT	y = 0.023 + 0.572x + 3.104x^2^ − 5.246x^3^	0.99	y = −0.963x^3^ + 0.775x^2^ + 0.997x − 0.005	0.99	12.38
BL	y = 0.009 + 0.3665x + 2.975x^2^ − 5.6016x^3^	0.99	y = −0.285x^3^ + 0.090x^2^ + 0.818x − 0.009	0.99	12.22
BT	y = 0.011 + 0.4145x + 3.331x^2^ − 6.32292x^3^	0.99	y = −1.439x^3^ + 1.032x2 + 0.738x − 0.004	0.99	9.90

## Data Availability

Data are contained within the article.

## Abbreviations

The following abbreviations are used in this manuscript:

CAGR	Compound annual growth rate
CTOD	Crack tip opening displacement
DIC	Digital image correlation
FEA	Finite element analysis
FPZ	Fracture process zone
LEFM	Linear elastic fracture mechanics
LLD	Load-line displacement
MAPE	Mean absolute percentage error
SENB	Single-edge notched bending
VCCT	Virtual crack closure technique
WPCs	Wood–plastic composites
XFEM	Extended finite element method

## References

[1] Ashby M.F. (1999). Materials Selection in Mechanical Design.

[2] Duruaku J.I., Okoye P.A.C., Onuegbu U.T., Onwukeme V.I., Okoye N.H., Nwadiogbu J.O. (2023). Physicomechanical properties of sustainable wood plastic composites of tropical sawdust and thermoplastic waste for possible utilization in the wood industry. Sustain. Bioenergy Syst..

[3] Androit Market Research. https://www.adroitmarketresearch.com/press-release/wood-plastic-composite-market.

[4] Grand View Research. Wood–Plastic Composites Market Size, Share and Trends Analysis Report by Product (Polyethylene, Polypropylene), by Application (Automotive Components), by Region, and Segment Forecasts, 2023–2030. Report ID: 978-1-68038-849-7, p. 198. https://www.grandviewresearch.com/industry-analysis/wood-plastic-compositesmarket#:~:text=The%20global%20wood%20plastic%20composites,11.6%25%20from%202023%20to%202030.

[5] Shahani S., Gao Z., Qaisrani M., Ahmed N., Yaqoob H., Khoshnaw F., Farooq S. (2021). Preparation and characterisation of wood plastic composites extracted from municipal waste. Polymers.

[6] Burgstaller C., Renner K. (2023). Recycling of wood–plastic composites—A reprocessing study. Macromol.

[7] Žiliukas A. (2006). Stiprumo ir Irimo Kriterijai.

[8] Clerk G., Brunner A.J., Niemz P., Van de Kuilen J.W. (2020). Application of fracture mechanics to engineering design of complex structures. 1st virtual European conference on fracture. Procedia Struct. Integr..

[9] Ritchie O.R., Liu D. (2021). Introduction to Fracture Mechanics.

[10] Liu Q., Liao Z., Cheng J., Xu D., Chen M. (2021). Mechanism of chip formation and surface-defects in orthogonal cutting of soft-brittle potassium dihydrogen phosphate crystals. Mater. Des..

[11] Anderson T.D. (2017). Fracture Mechanics: Fundamentals and Applications.

[12] Tschegg S.E.S., Navi P. (2009). Fracture behaviour of wood and its composites. A review. Holzforschung.

[13] Aicher S. (2010). Process zone length and fracture energy of spruce wood in mode-I from size effects. Wood Fiber Sci..

[14] Jungstedt E., Da Costa M.V.T., Östlund S., Berglund L.A. (2024). On the high fracture toughness of wood and polymer-filled wood composites—Crack deflection analysis for material design. Eng. Fract. Mech..

[15] (2018). Plastics–Determination of Fracture Toughness (G_IC_ and K_IC_)–Linear Elastic Fracture Mechanics (LEFM) Approach.

[16] Murray Y.D. (2007). Manual for LS-DYNA Wood Material Model 143.

[17] Ju S., Gross D., Hanenkamp N. (2022). Cutting force and vibration prediction of milling processes regarding the nonlinear behavior of cascade-controlled feed drives. Prod. Eng..

[18] (2014). Standard Test Method For Tensile Properties of Plastics.

[19] (2017). Standard Test Method for Compressive Properties of Rigid Plastics.

[20] (2009). Masyvios Medienos Skydai. Sanklijos Kokybė. Bandymo Metodas.

[21] (2014). Standard Test Methods for Plane–Strain Fracture Toughness and Strain Energy Release Rate of Plastic Materials.

[22] You Y., Zhang Y., Wang K., Zhou H. (2024). 3D–generalized maximum tangential strain criterion for predicting mixed-mode I/II/III fracture initiation of brittle materials considering T-stress effects. Theor. Appl. Fract. Mech..

[23] AZO Materials. A2 Tool Steel. https://www.azom.com/article.aspx?ArticleID=6218.

[24] Borrvall T. (2012). Mortar Contact for Implicit Analysis.

[25] Nakamura S., Suzuki S., Matsui K., Yamada T. FE evaluation of stress triaxiality/Lode angle dependencies of void growing process. Proceedings of the XV International Conference COMPLAS 2019 on Computational Plasticity, Fundamentals and Applications.

[26] Geshkova Z., Iliev V., Ivanov R. (2021). Influence on the degree of stress triaxiality and the Lode Angle on the fracture behavior of window frames. Sci. Eng. Educ..

[27] Liu X., Yan S., Rasmussen K.J.R., Deierlein G.G. (2022). Experimental investigation of the effect of Lode angle on fracture initiation of steels. Eng. Fract. Mech..

[28] Rahman Z.M. (2021). Mechanical and damping performance of flax fibre composites—A review. Compos. Part C.

[29] Young G.J. (2005). Fracture Behavior of Wood Plastic Composites (WPC). Master’s Thesis.

[30] Livermore Software Technology (LST) (2021). LS–DYNA Keyword User‘s Manual R13 Vol. II: Material Models.

[31] Chittajallu S.N.S.H., Richhariya A., Tse K.M., Chinthapenta V. (2022). A review on damage and rupture modelling for soft tissues. Bioengineering.

[32] Internal Energy LS-DYNA Support. https://www.dynasupport.com/howtos/general/internal-energy.

[33] University of Cambridge (2023). J-Shaped Curves. Dissemination of IT for the Promotion of Materails Science (DoITPoMS).

[34] Otkur A.M. (2010). Impact Modeling and Failure Modes of Composite Plywood. Master’s Thesis.

[35] Ricoeur A., Lindner F., Zarjov K. (2022). Stochastic aspects of crack deflection and crack path prediction in short fiber reinforced polymer matrix composites. Eur. J. Mech. A/Solids.

